# Is Rich and Rare the Common Share? Describing Biodiversity Patterns to Inform Conservation Practices for South American Anurans

**DOI:** 10.1371/journal.pone.0056073

**Published:** 2013-02-07

**Authors:** Fabricio Villalobos, Ricardo Dobrovolski, Diogo B. Provete, Sidney F. Gouveia

**Affiliations:** 1 Departamento de Ecologia, Instituto de Ciências Biológicas, Universidade Federal de Goiás, Goiânia, Goiás, Brazil; 2 Programa de Pós-Graduação em Ecologia e Evolução, Departamento de Ecologia, Instituto de Ciências Biológicas, Universidade Federal de Goiás, Goiânia, Goiás, Brazil; Consiglio Nazionale delle Ricerche (CNR), Italy

## Abstract

Species richness and range size are key features of biogeographic and macroecological analyses, which can yield a first assessment tool to define conservation priorities. Here we combined both features in a simultaneous analysis, based on range-diversity plots, to identify sets of rich-rare (high species richness with restricted ranges) and poor-rare cells (low species richness with restricted ranges). We applied this analysis to the anurans of South America and evaluated the representation of those sets of cells within the protected area system. South American anurans showed high species richness in the Brazilian Atlantic Forest and East Tropical Andes, while regions harboring most of the rare species were concentrated in the Andes and Atlantic Coast from North-Eastern Brazil to River Plate. Based on such patterns, we identified as rich-rare cells the Brazilian Atlantic Forest and Tropical Andes and as poor-rare cells the southern part of Andes and Uruguay. A low fraction of both sets of cells was represented within the protected area system. We show that a simultaneous consideration of species richness and rarity provides a rapid assessment of large-scale biodiversity patterns and may contribute to the definition of conservation priorities.

## Introduction

The ongoing biodiversity crisis claims for conservation actions that help mitigate it [1]. Limited availability of resources for conservation demands the definition of priorities [2]. Among these priorities, identifying particular regions of conservation value helps guiding resource allocation and avoiding competition with other potential land-uses [3]. The definition of spatial priorities must be based on an assessment of the geographic distribution of biodiversity features of conservation interest (e.g., numbers of species, phylogenetic and functional distinctiveness or genetic diversity) [4]–[7]. This geographic assessment of biodiversity features represents the first step towards informed conservation actions [4], [8].

Biogeography and macroecology aim to describe and explain spatial patterns of different biodiversity features. Results from biogeographical and macroecological approaches can provide the primary information for conservation assessments and planning [9]. Nonetheless, additional information (e.g., species' abundances, functional traits, phylogenetic relationships, ecosystem services, and region-specific sociopolitical issues) is still required for conducting detailed conservation actions [6]. Therefore, broad-scale conservation assessments constitute only an initial stage of planning from which more detailed assessments and prioritizations (e.g., at smaller spatial scales) can then be conducted [4].

Based on different biodiversity features, various procedures have been used to define spatial conservation priorities [10]. Commonly used criteria include the total number of species, of rare or narrow-ranged species, and the number of threatened species at a site or region [11], [12]. Species richness is a straightforward conservation target owing to its intrinsic significance for biodiversity definition and relative ease of monitoring [13]. Species' rarity, defined either by restricted geographic distribution or low population numbers, is of primary conservation concern owing to its relationship with species' threat status and extinction risk [14], thus the number of rare species is also used to establish spatial priorities.

Despite being relevant to virtually all conservation assessments and plans, species richness and rarity are seldom investigated simultaneously. Instead, they are used individually or comparatively to define different sets of species under study (e.g., all species *vs.* rare species richness) [12], [15]. Moreover, prioritizations based on either richness or rarity have been criticized owing to their emphasis on only one or two aspects of biodiversity (e.g., richness and threat) [16]. At broad spatial scales, species richness and rarity combined can provide a rapid assessment of biodiversity patterns for spatial priority setting. Recently, a macroecological framework that simultaneously considers richness and rarity, measured by range size, was introduced to describe geographic patterns of biodiversity with applications to conservation priority setting [17]–[20]. This framework uses primary biological information (i.e., species' presence-absence data) within range-diversity plots that describe the relationship between species richness and range size, providing a straightforward way to identify if species-rich and species-poor regions are composed mainly by rare or common (i.e., geographically restricted vs. widely distributed) species [20].

Here we applied such macroecological framework to describe geographic patterns of biodiversity and identified regions of potential conservation value in South America. Also, we used these regions to evaluate the performance of the protected area system. In doing so, we aimed to inform conservation practices by providing a broad-scale conservation assessment that could be integrated into more detailed prioritizations. For this, we used amphibians as our case study. Amphibians are a particularly threatened vertebrate group, with *ca.* 32% of species undergoing a combination of hazards including habitat loss, climate change and emergent diseases [21]–[25]. Their vulnerability is boosted by their overall smaller range sizes, relative to other terrestrial vertebrates [15], which has been shown to correlate with amphibian threat status and extinction risk [25]. Most threatened amphibians are anurans (frogs and toads), which are the most species-rich and geographically widespread amphibian order [26]. Anuran biodiversity, including three of the main conservation aspects (species richness, rarity, and threat), is concentrated over tropical regions, especially in South America [25], [26], making this a critical region for amphibian conservation.

## Materials and Methods

We mapped the geographic distribution of South American anurans (i.e., extent of occurrence) drawn from the Global Amphibian Assessment [22] onto a 1°×1° resolution grid. From this grid, we built a presence-absence matrix (PAM) of 1520 cells by 2437 species. The PAM is a binary matrix representing the basic biogeographical information on species richness and occurrence over a particular region [27]. We followed Arita et al.'s [17] *Qr-mode* to gather information on the number and mean range size of species present at each cell from the PAM. This procedure extracts data from rows (i.e., grid cells: species richness) by simultaneously considering the information within columns (i.e., species: range size). This information can be summarized and depicted using range-diversity (RD) plots [17], [18].

RD plots convey joint information on species richness and range size enabling the exploration of biogeographic patterns of similarity among grid cells, in terms of shared species, and co-occurrence among species [18]. We used the “by sites” version of RD plots [17], in which axes represent the mean proportional per-cell range size and the proportional species richness of cells, in the *x*- and *y*-axes, respectively. Central tendency of points within the RD plot by sites is determined by the average proportional range size of the whole system depicted by a vertical, dashed line; whereas detailed point dispersion depends on the overall covariance among cells resulting from the number of cells with which each individual cell shares its species [17]. In general, points located to the left of the plot's vertical line depict cells with negative average covariances (i.e., on average, sharing none or few species with most cells) whereas points to the right indicate cells with positive average covariances (i.e., on average, sharing many species with most cells). RD plots and associated parameters were obtained using the script available from [18] for the R statistical language [28].

We applied a quantile approach to define subsets of cells with different combinations of richness and range size values within the RD plot [20]. We defined “rich-rare” cells as those lying within the fourth quartile of species richness (i.e., cells with higher proportional richness) and first quartile of range size values (i.e., cells with lower mean range size). Additionally, we defined “poor-rare” cells as those falling within the first quartiles of species richness (i.e., cells with lower proportional richness) and range size values. This quantile approach is a pragmatic criterion based solely on the frequency distribution of species richness and range size values of the study system, which has been commonly used to define species richness hotspots (e.g., [11], [12]) or species' rarity (e.g., [15], [29]). We mapped the geographic pattern of rich-rare and poor-rare cells, along with the overall pattern of anuran species richness and per-cell range size within South America.

We used the World Database on Protected Areas [30] to define protected grid-cells throughout South America. Protected cells were defined as those grid-cells with more than 10% of its area covered by protected areas (categories I–IV of IUCN). These protected cells were used to evaluate the performance of the South American protected area system in representing rich-rare and poor-rare cells. Significant spatial congruencies between our rich-rare and poor-rare cells and protected cells were tested using randomization tests. We quantified the observed spatial overlaps by counting the number of protected cells falling within our defined sets (i.e., observed value). Then we estimated the significance of these spatial overlaps by randomizing the position of protected cells (999 times) and recalculating the spatial overlaps between these and our rich-rare and poor-rare cells (i.e., random values). The proportion of randomized values equal or superior to the observed value was considered as the *P*-value of the randomization test. The null hypothesis that the observed overlap is lower than expected by chance was tested at the 5% significance level. Statistical analyses were done in R [28].

## Results

**Figure 1:**
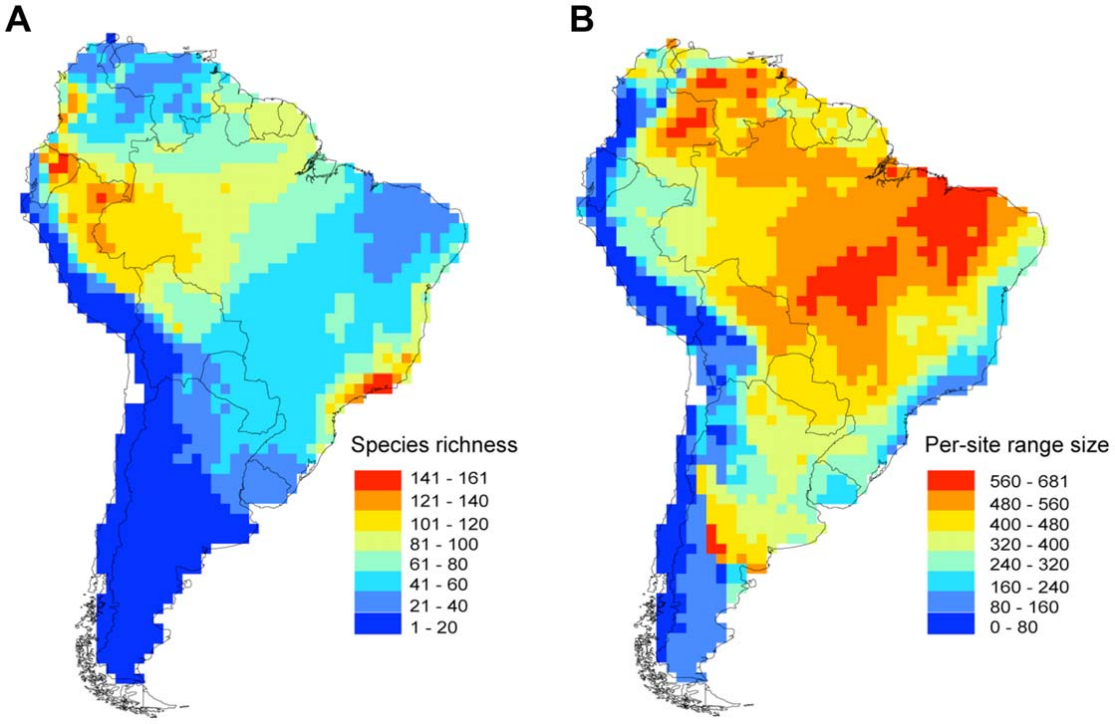
Geographic patterns of anuran biodiversity in South America. Maps depicting (A) species richness and (B) per-cell range size.

Anuran species richness varied widely, from one to 161 species among cells (53.83±34.20, mean ± SD). Regions with the highest richness were the eastern tropical Andes, Amazonian basin, and the Brazilian Atlantic Forest (Fig. 1A). Rarity regions (i.e., low mean per-cell range size) concentrated in the tropical Andes and the Atlantic coast from North-Eastern Brazil to River Plate, but also in the southern Andes along the Austral temperate forests. These regions included both species-rich and species-poor cells (Fig. 1B).

**Figure 2:**
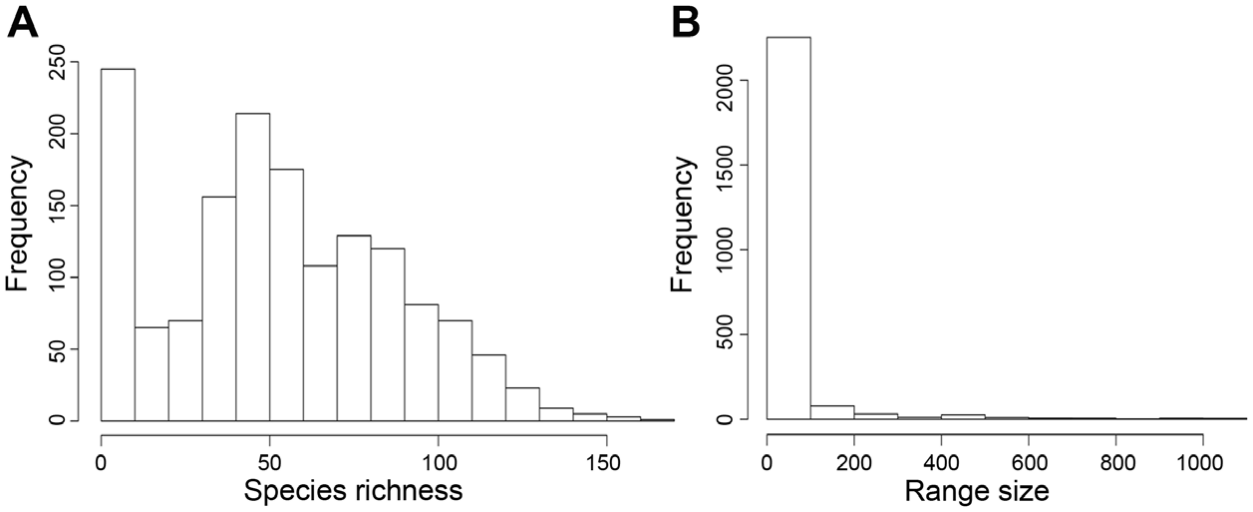
Histograms of (A) species richness and (B) range size frequency distributions.

Species richness frequency distribution (i.e., the distribution of richness values over all cells) showed a bimodal pattern, peaking at low and intermediate richness values with few cells having high species richness (Fig. 2A). The vast majority of anurans (97.7% of species) occupied less than one quarter of South America and only a few species (0.6% of species) occurred in more than 50% of the continent (mean = 33.6 grid-cells, SD = 110.2). In fact, 35.7% of species occurred in only one grid-cell. This pattern yielded a highly right-skewed range size frequency distribution; with far more species with small range sizes than species with large ranges (Fig. 2B).

**Figure 3:**
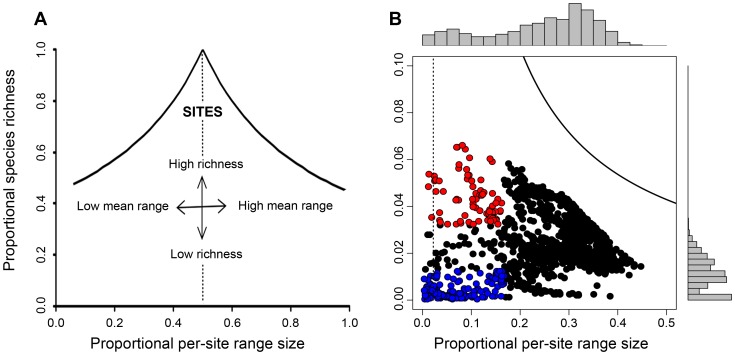
Range-Diversity (RD) plot by sites of South American anurans. (A) Idealized RD plot showing the theoretical boundaries (solid line), average value of mean proportional range size (vertical, dashed line) and interpretation of point dispersion (redrawn from [20] with permission from Elsevier). (B) Observed RD plot depicting the rich-rare (red dots) and poor-rare cells (blue dots). Histograms on top and on the right-hand side of (B) show the frequency distribution of *x*-axis and *y*-axis variables, respectively.

The RD plot lacked a clear internal structure, with points arranged towards the lower left corner of the plot but with considerable dispersion along the *x*-axis (Fig. 3B). Most cells showed positive covariance, indicating that they generally share species (Fig. 3B). Nevertheless, there was a high turnover of species as shown by the Whittaker's beta (β*_w_* = 45.27), whose reciprocal value (1/β*_w_* = 0.022) equals the average proportional species richness and the average proportional range size in the system [17]. This means that cells contained, on average, 2.2% of species; and that the average anuran species occurred in 2.2% of cells.

**Figure 4:**
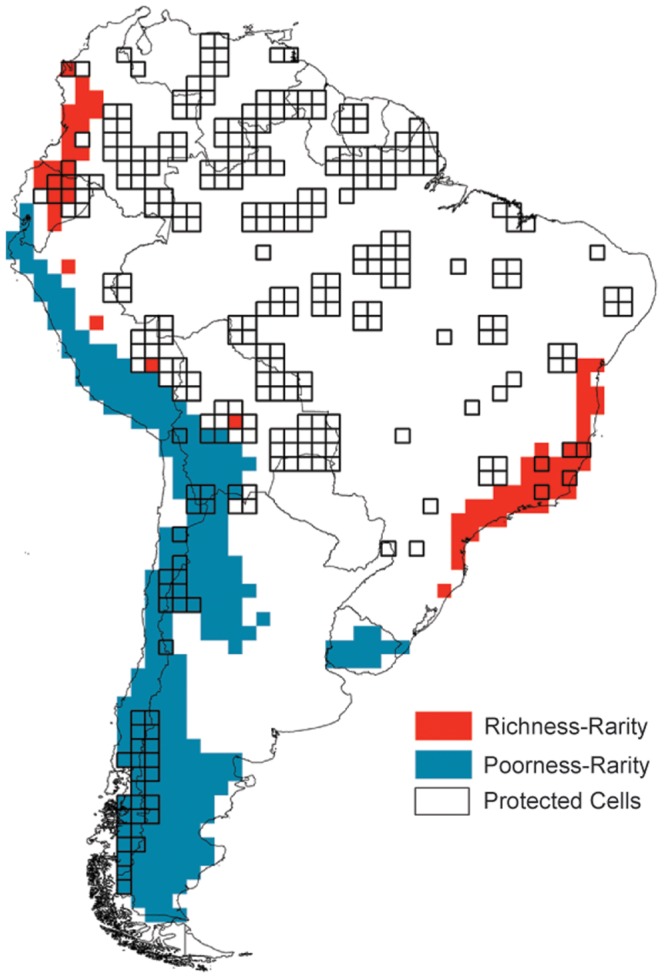
Rich-rare and poor-rare sets of cells within South America, depicting the location of protected cells.

The rich-rare set comprised 71 cells harboring more than 79 species and averaging less than 254 cells in per-cell range size. Rich-rare cells were distributed almost exclusively in mountainous areas over the tropical Andes and the Brazilian Atlantic Forest. Conversely, the poor-rare set comprised 255 cells harboring less than 31 species and averaging less than 254 cells in per-cell range size. Poor-rare cells were located mainly over the western-central and southern regions of South America along the southern Andes but also in Uruguay (Fig. 4).

Rich-rare and poor-rare cells were poorly represented by the protected area system within South America (Fig. 4). Less than 25% of cells within both rich-rare and poor-rare sets (21.13% and 16.47% of cells, respectively) contained at least one protected cell within them. This pattern was not significantly different from random placements of the observed number of protected areas within South America (*P* = 0.463 and *P* = 0.944 for the rich-rare and poor-rare sets, respectively).

## Discussion

We presented the first comprehensive analysis of anuran biodiversity patterns throughout South America simultaneously considering two of the most important biodiversity features: species richness and range size. This biogeographic description allowed us to identify regions within South America that may be relevant for conservation of anuran biodiversity. For instance, these identified regions can be used as geographical templates where further attention can be directed.

Species richness can be positively related with species rarity when cells occupied by rare species are also species-rich and represent a subset of cells occupied by widespread species [18], [20]. Our results showed that rare anuran species occurred at both species-rich and species-poor regions within South America. Regions with the highest species richness, namely the equatorial Andes and the Brazilian Atlantic Forest, tended to have species averaging small range sizes, whereas regions with intermediate species richness like eastern Amazonia and the Cerrado biome (i.e., Brazilian Savanna) harbored species averaging large ranges. Interestingly, regions with low species richness also presented small mean range sizes, especially those at the southern tip and western-central South America. These latter findings are in contrast with the common assumption that restricted or endemic species are located mainly in species-rich regions [31], [32].

The partial, positive relationship between richness and rarity found for South American anurans could be highly informative to prioritize regions for conservation of this vertebrate group. For instance, geographic congruence between high richness and high rarity regions could guarantee the protection of rare species if only the richest cells were selected for conservation. Indeed, previous studies have supported this richness-rarity relationship. For example, global analyses of terrestrial vertebrates showed that regions with high richness of narrow-ranging species were congruent with species-rich regions, suggesting that rarity could be used as a surrogate for the conservation of overall species richness [15], [31]. Moreover, results from those analyses were similar for different taxonomic groups, supporting the role of indicator groups in which richness of rare species from one taxonomic group correlates positively with overall richness of other taxonomic groups (e.g., [31], [32]). Accordingly, anurans seem to be a good indicator of biodiversity patterns of other terrestrial vertebrates (e.g., [32]). Therefore, the observed richness-rarity patterns we found could facilitate spatial prioritization for South American vertebrates in general.

Conversely, the observed relationship between species-poor regions and rarity for South American anurans challenges conservation actions. Many cells held low numbers of anuran species with small range sizes. Such cells represent unique assemblages usually not incorporated into common conservation actions focused solely on species richness. Consequently, these species-poor cells or “coldspots” [16] may be left out of conservation actions, though they are certainly relevant. Indeed, more comprehensive approaches do consider such species-poor cells [6], [33]. For instance, under a complementarity criterion, if a cell holds a low number of species that do not occur elsewhere, this cell will be important in determining the network of cells needed to protect all species [6], [7], [34]. Yet, our simple approach considering both species richness and range size at large spatial scales would help identifying such coldspots in a straightforward manner.

Identification of poor-rare cells may also point towards particular ecosystems or habitats usually downplayed in most conservation strategies owing to their limited species diversity [16]. Poor-rare cells for anurans in South America concentrated at the southern tip and western-central regions, which are covered by temperate forests and steppes. These vegetation types are indeed poorer in species richness when compared to tropical forests. Nevertheless, the presence of species restricted to those temperate forests and steppes habitats highlight their relevance for biodiversity conservation. In addition, following the reasoning of indicator groups, it is possible that coldspots for one taxonomic group be so for other taxonomic groups too. This possibility could be evaluated in the same manner as the identification of species-rich regions for different taxa [15], [29], [31].

Recent studies have highlighted the importance of Neotropical ecoregions for the conservation of terrestrial vertebrates, including anurans [35]–[37]. For example, Loyola et al. [35] identified a set of 49 ecoregions that if sufficiently covered by reserves would retain at least 80% of total, endemic and threatened vertebrate species altogether. Some of their priority ecoregions are spatially congruent with our rich-rare and poor-rare cells. For instance, our rich-rare cells were located along the Serra do Mar Coastal forests and Chocó-Darien moist forests, whereas our poor-rare cells fell within the Patagonian steppe, Valdivian temperate forests and Uruguayan savanna. The congruence between Loyola et al. [35] results and ours is not surprising because theirs relied on a complementarity criterion that is bound to select regions with rare species even if they are species poor. Instead, such spatial congruence highlights the advantage of our simple approach for conducting initial assessments of priority regions.

The current protected area system in South America fails to encompass important regions for the conservation of anurans. For instance, large regions with few rare anuran species along the southern Andes in western-central South America and central Uruguay are virtually lacking protected areas. These coldspots would be overlooked by protected area systems if conservation assessments fail to apply more integrative approaches. Urbina-Cardona and Loyola [38] obtained similar findings for the representation of endangered hylid frogs (tree-frogs) within the Neotropical protected areas, showing that most restricted-range tree-frogs have only the periphery of their distributions under protection [38]. Our findings and those of other authors (e.g., [38]) on the scarce protection of South American anuran biodiversity are not surprising. Instead, these results highlight the inefficiency of the *ad hoc* manner of establishing protected areas to preserve biodiversity of specific taxa [39], [40].

Aside from providing objective planning frameworks [9], the application of biogeographic methods and theory allow pattern interpretation and inference of potential processes responsible for such patterns [41], [42]. For instance, amphibian biodiversity patterns have been causally related to current environmental conditions (e.g., energy: water and temperature; [43], [44]) but also to historical processes (e.g., historical climate oscillations, time for speciation, niche conservatism; [45], [46]). Likewise, rich-rare and poor-rare regions identified by our approach can be interpreted as a result of historical processes. For example, processes like lineage origin and speciation followed by extinction within a region or colonization of few lineages followed by low speciation could have given rise to poor-rare assemblages [47]. Conversely, rich-rare regions could be the result of high speciation coupled with low extinction rates [47]. Evaluating the phylogenetic structure of assemblages [48] within rich-rare and poor-rare regions could provide tests for such hypotheses. For example, if the aforementioned processes hold, we could expect phylogenetic clustering (i.e., presence of closely related species) in poor-rare regions and phylogenetic overdispersion (i.e., presence of distantly related species) in rich-rare regions.

## Conclusions

Our approach described complex biogeographic patterns of South American anurans to inform conservation prioritization. Other biological data are undoubtedly necessary (e.g., species' traits, phylogenetic relationships) for a proper establishment of conservation priorities, but these are difficult to obtain and not always available. Thus, we believe that our spatial assessment of broad-scale biodiversity patterns of South American anurans will prove useful to inform future conservation planning and practices based on current knowledge for this threatened vertebrate group.

## References

[1] Hoffmann M, Hilton-Taylor C, Angulo A, Böhm M, Brooks TM, et al (2010) The impact of conservation on the status of the world's vertebrates. Science 330: 1503–1509. 20978281 10.1126/science.1194442

[2] Moilanen A, Arponen A (2011) Setting conservation targets under budgetary constraints. Biol Conserv 144: 650–653.

[3] Wilson KA, Underwood EC, Morrison SA, Klausmeyer KR, Murdoch WW, et al (2007) Conserving biodiversity efficiently: what to do, where, and when. PLoS Biol 5 (9) e223. 17713985 10.1371/journal.pbio.0050223 PMC1950771

[4] Knight AT, Driver A, Cowling RM, Maze K, Desmet PG, et al (2006) Designing systematic conservation assessments that promote effective implementation: best practice from South Africa. Conserv Biol 20: 739–750. 16909567 10.1111/j.1523-1739.2006.00452.x

[5] Devictor V, Mouillot D, Meynard C, Jiguet F, Thuiller W, et al (2010) Spatial mismatch and congruence between taxonomic, phylogenetic and functional diversity: the need for integrative conservation strategies in a changing world. Ecol Lett 13: 1030–1040. 20545736 10.1111/j.1461-0248.2010.01493.x

[6] Margules C, Sarkar S (2007) Systematic Conservation Planning. Cambridge: Cambridge University Press. 278 p.

[7] Margules C, Pressey R, Williams P (2002) Representing biodiversity: data and procedures for identifying priority areas for conservation. J Biosci 27: 309–326. 12177531 10.1007/BF02704962

[8] Knight AT, Cowling RM, Boshoff AF, Wilson SL, Pierce SM (2011) Walking in STEP: Lessons for linking spatial prioritisations to implementation strategies. Biol Conserv 144: 202–211.

[9] Whittaker RJ, Araújo MB, Paul J, Ladle RJ, Watson JEM, et al (2005) Conservation biogeography: assessment and prospect. Divers Distrib 11: 3–23.

[10] Cabeza M, Moilanen A (2001) Design of reserve networks and the persistence of biodiversity. Trends Ecol Evol 16: 242–248. 11301153 10.1016/s0169-5347(01)02125-5

[11] Ceballos G, Ehrlich PR (2006) Global mammal distributions, biodiversity hotspots and conservation. Proc Natl Acad Sci USA 103: 19374–19379. 17164331 10.1073/pnas.0609334103 PMC1698439

[12] Orme CDL, Davies RG, Burgess M, Eigenbrod F, Pickup N, et al (2005) Global hotspots of species richness are not congruent with endemism or threat. Nature 436: 1016–1019. 16107848 10.1038/nature03850

[13] Magurran AE (1988) Ecological diversity and its measurement. Princeton: Princeton University Press. 192 p.

[14] Gaston KJ (1994) Rarity. London: Chapman & Hall. 220 p.

[15] Grenyer R, Orme CDL, Jackson SF, Thomas GH, Davies RG, et al (2006) Global distribution and conservation of rare and threatened vertebrates. Nature 44: 93–96. 10.1038/nature05237 17080090

[16] Kareiva P, Marvier M (2003) Conserving biodiversity coldspots. Am Sci 91: 344–351.

[17] Arita HT, Christen JA, Rodríguez P, Soberón J (2008) Species diversity and distribution in presence-absence matrices: mathematical relationships and biological implications. Am Nat 112: 519–532. 10.1086/590954 18729736

[18] Arita HT, Christen JA, Rodríguez P, Soberón J (2012) The presence-absence matrix reloaded: the use and interpretation of range-diversity plots. Glob Ecol Biogeogr 21: 282–292.

[19] Soberón J, Ceballos G (2011) Species Richness and Range Size of the Terrestrial Mammals of the World: Biological Signal within Mathematical Constraints. PLoS One 6 (5) e19359. 21573112 10.1371/journal.pone.0019359 PMC3089617

[20] Villalobos F, Lira-Noriega A, Soberón J, Arita HT (2012) Range-diversity plots for conservation assessments: using richness and rarity in conservation priority setting. Biol Conserv 158: 313–320.

[21] Hof C, Araújo MB, Jetz W, Rahbek C (2011) Additive threats from pathogens, climate and land-use change for global amphibian diversity. Nature 480: 516–521. 22089134 10.1038/nature10650

[22] IUCN, Conservation International, NatureServe (2009) Global Amphibian Assessment. Available: http://www.globalamphibians.org. Accessed 2012 Sep 4.

[23] Ochoa-Ochoa LM, Rodríguez P, Mora F, Flores-Villela O, Whittaker RJ (2012) Climate change and amphibian diversity patterns in Mexico. Biol Conserv 150: 94–102.

[24] Stuart SN, Chanson JS, Cox NA, Young BE, Rodrigues ASL, et al (2004) Status and trends of amphibian declines and extinctions worldwide. Science 306: 1783–1786. 15486254 10.1126/science.1103538

[25] Cooper N, Bielby J, Thomas GH, Purvis A (2008) Macroecology and extinction risk correlates of frogs. Glob Ecol Biogeogr 17: 211–221.

[26] Duellman WE, Trueb L (1994) Biology of amphibians. Baltimore: Johns Hopkins University Press. 670 p.

[27] Gotelli NJ, Anderson MJ, Arita HT, Chao A, Colwell RK, et al (2009) Patterns and causes of species richness: a general simulation model for macroecology. Ecol Lett 12: 873–886. 19702748 10.1111/j.1461-0248.2009.01353.x

[28] R Development Core Team (2012) R: a language and environment for statistical computing. R Foundation for Statistical Computing, Vienna, Austria.

[29] Orme CDL, Davies RG, Olson VA, Thomas GH, Ding TS, et al (2006) Global patterns of geographic range size in birds. PLoS Biol 4 (7) e208. 16774453 10.1371/journal.pbio.0040208 PMC1479698

[30] WDPA Consortium (2009) World Database on Protected Areas. Available: http://www.unep-wcmc.org/wdpa/index.htm. Accessed 2009 Mar 9.

[31] Lamoreux JF, Morrison JC, Ricketts TH, Olson DM, Dinerstein E, et al (2006) Global tests of biodiversity concordance and the importance of endemism. Nature 440: 212–214. 16382239 10.1038/nature04291

[32] Loyola RD, Kubota U, Lewinsohn TM (2007) Endemic vertebrates are the most effective surrogates for identifying conservation priorities among Brazilian ecoregions. Divers Distrib 13: 389–396.

[33] Sarkar S, Pressey RL, Faith DP, Margules CR, Fuller T, et al (2006) Biodiversity conservation planning tools: present status and challenges for the future. Annu Rev Environ Resour 31: 123–159.

[34] Vane-Wright RI, Humphries CJ, Williams PH (1991) What to protect? — Systematics and the agony of choice. Biol Conserv 55: 235–254.

[35] Loyola RD, Kubota U, da Fonseca GAB, Lewinsohn TM (2009) Key Neotropical ecoregions for conservation of terrestrial vertebrates. Biodivers Conserv 18: 2017–2031.

[36] Diniz-Filho JAF, Bini LM, Pinto MP, Rangel TFLVB, Carvalho P, et al (2006) Anuran species richness, complementarity and conservation conflicts in Brazilian Cerrado. Acta Oecol 29: 9–15.

[37] Loyola RD, Becker CG, Kubota U, Haddad CFB, Fonseca CR, et al (2008) Hung out to dry: choice of priority ecoregions for conserving threatened Neotropical anurans depends on life-history traits. PLoS One 3 (5) e2120. 18461175 10.1371/journal.pone.0002120 PMC2361192

[38] Urbina-Cardona JN, Loyola RD (2010) Applying niche-based models to predict endangered-hylid potential distributions: are neotropical protected areas effective enough? Trop Conserv Sci 1: 417–445.

[39] Rodrigues ASL, Andelman SJ, Bakarr MI, Boitani L, Brooks TM, et al (2004) Effectiveness of the global protected area network in representing species diversity. Nature 428: 640–643. 15071592 10.1038/nature02422

[40] Pressey R (1994) Ad hoc reservations: forward or backward steps in developing representative reserve systems? Conserv Biol 8: 662–668.

[41] Kerr JT, Kharouba HM, Currie DJ (2007) The macroecological contribution to global change solutions. Science 316: 1581–1584. 17569854 10.1126/science.1133267

[42] Ladle RJ, Whittaker RJ (2011) Conservation biogeography. Oxford: Wiley-Blackwell. 320 p.

[43] Buckley LB, Jetz W (2008) Linking global turnover of species and environments. Proc Natl Acad Sci USA 105: 17836–17841. 19001274 10.1073/pnas.0803524105 PMC2584760

[44] Whitton FJS, Purvis A, Orme CDL, Olalla-Tárraga MÁ (2012) Understanding global patterns in amphibian geographic range size: does Rapoport rule? Glob Ecol Biogeogr 21: 179–190.

[45] Araújo MB, Nogués-Bravo D, Diniz-Filho JAF, Haywood AM, Valdes PJ, et al (2008) Quaternary climate changes explain diversity among reptiles and amphibians. Ecography 31: 8–15.

[46] Gouveia SF, Hortal J, Cassemiro FAS, Rangel TF, Diniz-Filho JAF (2012) Nonstationary effects of productivity, seasonality, and historical climate changes on global amphibian diversity. Ecography (doi:10.1111/j.1600-0587.2012.07553.x).

[47] Jablonski D, Roy K, Valentine JW (2006) Out of the Tropics: Evolutionary Dynamics of the Latitudinal Diversity Gradient. Science 314: 102–106. 17023653 10.1126/science.1130880

[48] Webb CO, Ackerly DD, McPeek MA, Donoghue MJ (2002) Phylogenies and community ecology. Annu Rev Ecol Syst 33: 475–505.

